# H-RN, a peptide derived from hepatocyte growth factor, inhibits corneal neovascularization by inducing endothelial apoptosis and arresting the cell cycle

**DOI:** 10.1186/1471-2121-14-8

**Published:** 2013-02-24

**Authors:** Ye Sun, Li Su, Zhongxiao Wang, Yi Xu, Xun Xu

**Affiliations:** 1 Department of Biology, Shanghai Institute of Technology, 200235, Shanghai, P.R. China; 2 Department of Ophthalmology, Shanghai First People’s Hospital, Shanghai JiaoTong University, Haining Road 100, 200080, Shanghai, P.R. China

**Keywords:** H-RN, Peptide, HUVECs, Cornea, Neovascularization

## Abstract

**Background:**

The goal of this study was to investigate the anti-angiogenic activity of a novel peptide H-RN, derived from the hepatocyte growth factor kringle 1 domain (HGF K1), in a mouse model of corneal neovascularization. The anti-angiogenic effect of H-RN on vascular endothelial growth factor (VEGF)-stimulated cell proliferation, cell migration and endothelial cell tube formation was assessed *in vitro* using Human Umbilical Vein Endothelial Cells (HUVECs) and *in vivo* using a mouse cornea micropocket assay. Apoptosis and cell cycle arrest were assessed by flow cytometry. A scrambled peptide was used as a negative control.

**Results:**

H-RN effectively inhibited VEGF-stimulated HUVEC proliferation, migration and tube formation on Matrigel, while a scrambled peptide exerted no effect. In the mouse model of corneal angiogenesis, VEGF-stimulated angiogenesis was significantly inhibited by H-RN compared to a scrambled peptide that had no such activity. VEGF protected HUVECs from apoptosis, while H-RN inhibited this protective effect of VEGF. VEGF significantly increased the proportion of cells in the S phase compared to control treated cells (*p*<0.05). Treatment with H-RN (1.5 mM) induced the accumulation of cells in G0/G1 phase, while the proportion of cells in the S phase and G2/M phase decreased significantly compared to control group (*p*<0.05).

**Conclusions:**

H-RN has anti-angiogenic activity in HUVECs and in a mouse model of VEGF-induced corneal neovascularization. The anti-angiogenic activity of H-RN was related to apoptosis and cell cycle arrest, indicating a potential strategy for anti-angiogenic treatment in the cornea.

## Background

The transparency and refraction properties of a normal, healthy cornea are predominantly mediated by its avascularity. However, many pathological processes affecting the cornea, including trauma, inflammation, infection, toxicity and nutritional insults, may lead to corneal neovascularization [1]. While this process may be beneficial in wound healing, clearance of infections and arresting progressive immune-mediated corneal melts, neovascularization may reduce corneal transparency by growing into the normally vascular-free cornea tissue, bleeding into the cornea and causing lipid deposition, tissue swelling and scarring [2]. Corneal neovascularization may significantly impair visual acuity and the immune privilege of the cornea, and may also worsen the prognosis of corneal transplantation [3-6]. The prevalence of corneal neovascularization ranges from 125,000-470,000 individuals in the population wearing soft contact lenses [7] and is associated with the second most common cause of blindness globally, and with the most common form of corneal blindness in developed countries [8].

Clinical ophthalmologists have been challenged with the treatment of sight-threatening corneal neovascularization for over 100 years [8]. However, because age-related maculopathy is the major cause of blindness in the aged population in developed world, the anterior segment neovascularization inhibition still lags behind anti-angiogenesis at the posterior pole of the eye [8]. The current clinical treatment for corneal angiogenesis primarily involves direct anti-angiogenesis agents (bevacizumab, ranibizumab and pegaptanib), indirect anti-angiogenesis agents (anti-inflammatory steroids and cyclosporine A) [8,9], laser photocoagulation, fine-needle diathermy, photodynamic therapy [10] and conjunctival, limbal or amniotic membrane transplantation. However, regular application of corticosteroids may increase the risk of infection and induce cataracts and glaucoma [9]. Other methods of treatment often require multiple treatment sessions with risks of severe complication arising, since significantly more laser energy is needed than that used for the treatment of choroidal neovascularization. Furthermore, photodynamic therapy, which is reported to significantly inhibit corneal neovascularization, only blocks existing neovascularization, but does not prevent pathological angiogenesis. Currently, treatments targeting the molecular mediators of angiogenesis are widely studied and represent the ideal choice for therapy. VEGF is a major mediator of the angiogenic process. Numerous therapeutic strategies targeting VEGF are being studied [11,12]. New therapeutic agents, including pegaptanib, ranibizumab and particularly bevacizumab, are reported to be effective in inhibiting corneal neovascularization. However, the extremely high annual treatment cost of these agents has limited their widespread use and systemic adverse events have been reported [13,14].

The design and development of peptides to inhibit angiogenesis is an important area in anti-angiogenic drug development [15]. In comparison to proteins, peptides display lower immunogenicity, higher solubility in water, stable production methods, improved consistency between batches and are superior at targeting and penetrating tumors [15]. Previously, we identified a novel peptide, H-RN, derived from the hepatocyte growth factor kringle 1 domain (HGF K1), and demonstrated that it has anti-angiogenic activity to retinal neovascularization in a mouse model [16]. In the current study, we further investigated the ability of H-RN to inhibit corneal neovascularization and possible anti-angiogenesis mechanisms. Our study may lead to new potential drug discoveries and the development of novel treatments for pathological retinal and corneal angiogenesis.

## Results

### H-RN inhibits HUVEC proliferation

**Figure 1:**
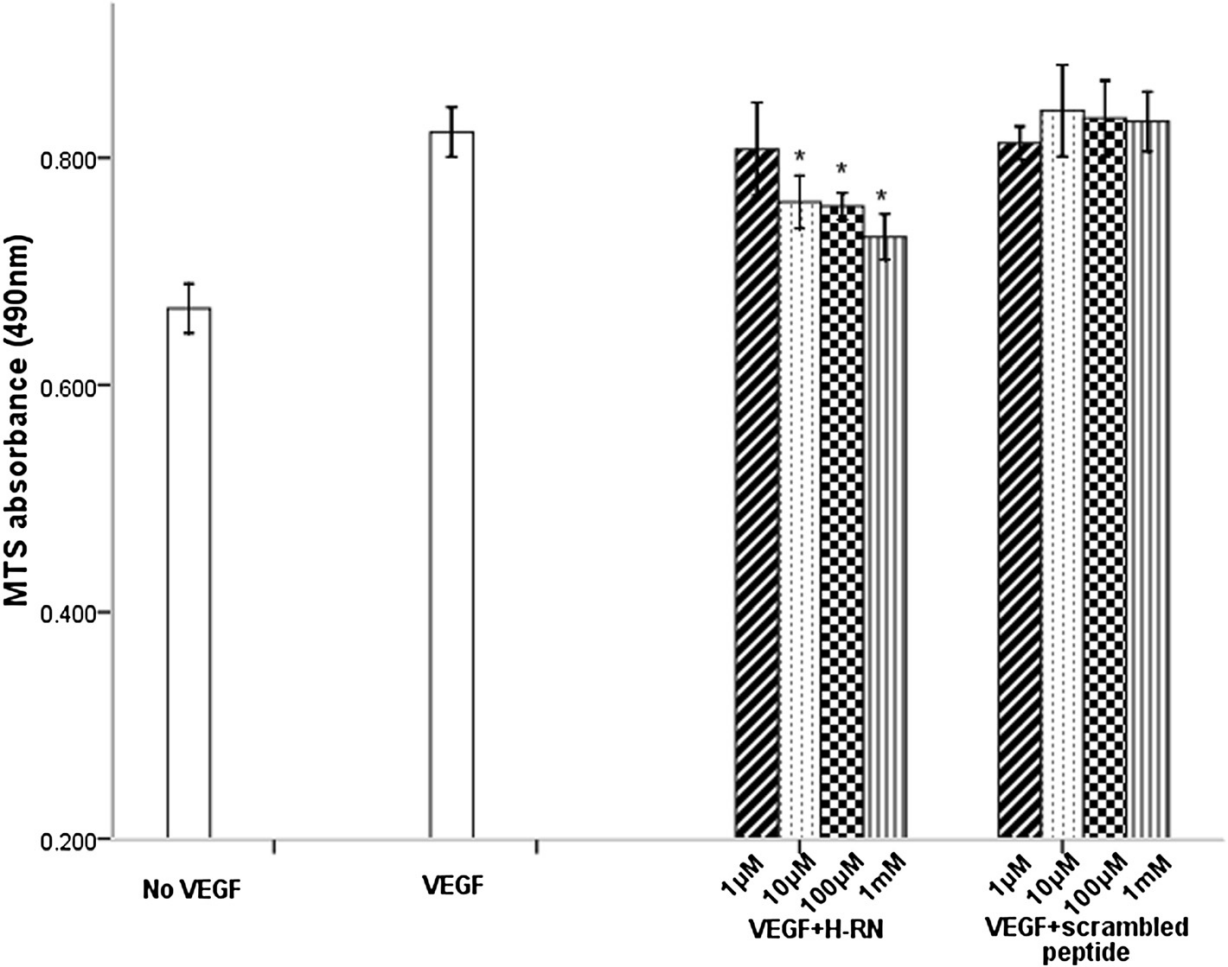
**H-RN inhibits human umbilical vein endothelial cell (HUVEC) proliferation while scrambled peptide exerts no effect.** Following starvation, HUVECs were incubated with vascular endothelial growth factor (VEGF) (100 ng/ml) and various concentrations of H-RN or scrambled peptide for 24 h. Cell proliferation was significantly inhibited by H-RN, especially at 1 mM. Scrambled peptide had no inhibitory activity on HUVECs at various concentrations (*p<0.05, each condition versus VEGF group).

To investigate the effect of H-RN on HUVEC proliferation, cells were exposed to VEGF (100 ng/ml) alone and in combination with various concentrations of H-RN or scrambled peptide for 24 h. In comparison with control cells, treatment with VEGF alone induced a significant increase in cell proliferation (Figure 1, *p*<0.05). Treatment of cells with increasing concentrations of H-RN peptide resulted in a significant inhibition of cell proliferation (*p*<0.05), reaching maximal inhibition at 1 mM H-RN. In contrast, treatment of cells with scrambled peptide had no inhibitory effect (Figure 1, *p*>0.05). These results indicate that H-RN impairs VEGF-induced HUVEC proliferation.

### HUVECs migration towards VEGF is inhibited by H-RN

**Figure 2:**
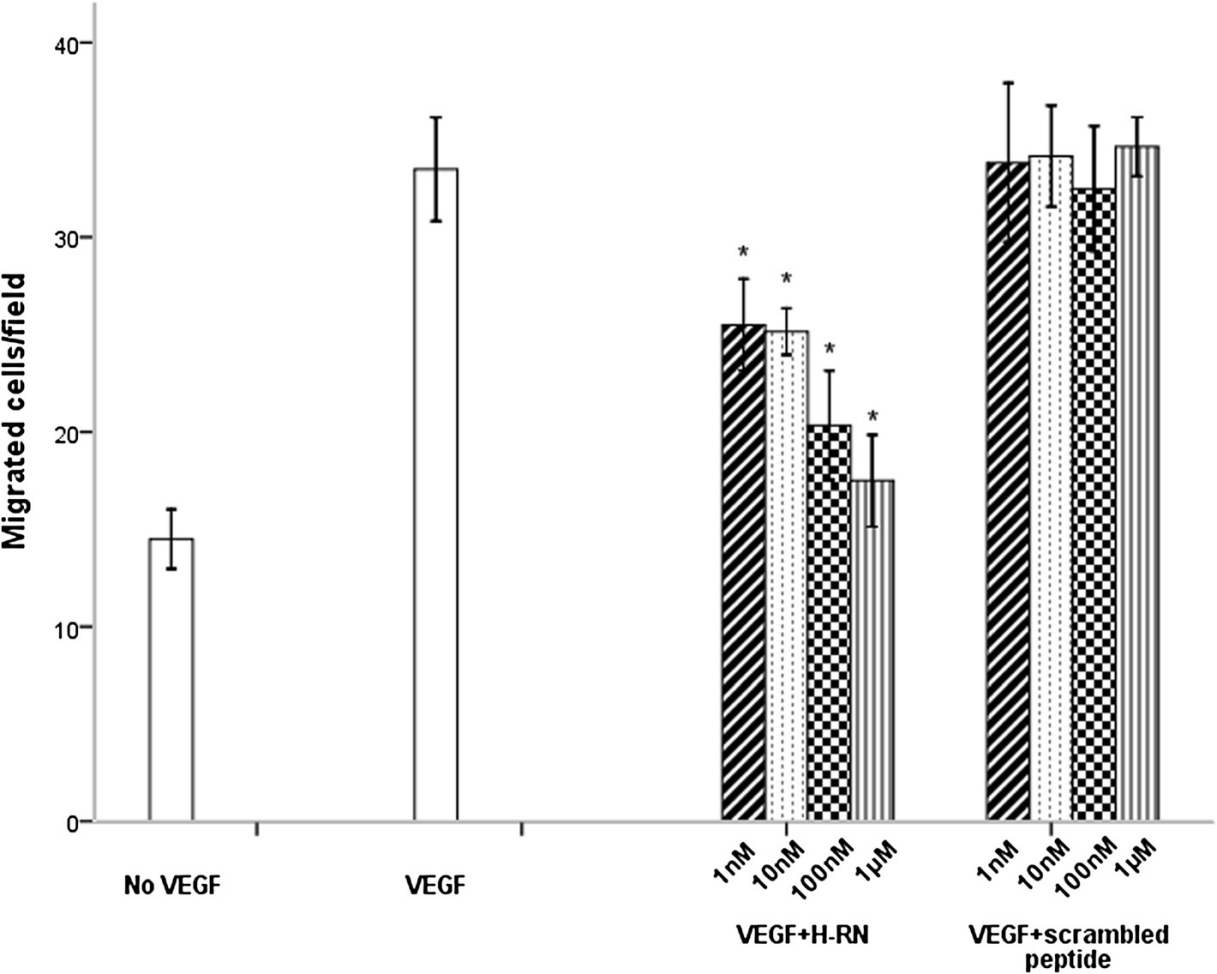
**Effect of peptides on the migration of HUVECs stimulated by VEGF.** HUVECs were pre-incubated with different concentrations of peptides in the upper chamber for 30 min before stimulation with VEGF (100 ng/ml) in the lower chamber. After incubation for 24 h, the migratory activity of cells was estimated. H-RN inhibited migration of HUVECs. Scrambled peptide did not inhibit migration up to 1 μM. (*p<0.05; each condition versus VEGF group).

Next, we investigated the ability of H-RN to affect the migration of HUVECs using Transwell migration assays. Compared to control, VEGF significantly stimulated HUVEC migration through the porous membrane of Transwell chambers (*p*<0.001). Various concentrations of H-RN (1 nM, 10 nM, 100 nM and 1 μM) were then added to test the inhibitory effect of this peptide on VEGF-induced HUVECs migration. Scrambled peptide was added as control. The results showed that H-RN significantly inhibited cell migration, especially at 100 nM and 1 μM (*p*<0.001) compared with control scrambled peptide (*p*>0.05) (Figure 2).

### H-RN inhibits HUVEC tube formation in Matrigel

**Figure 3:**
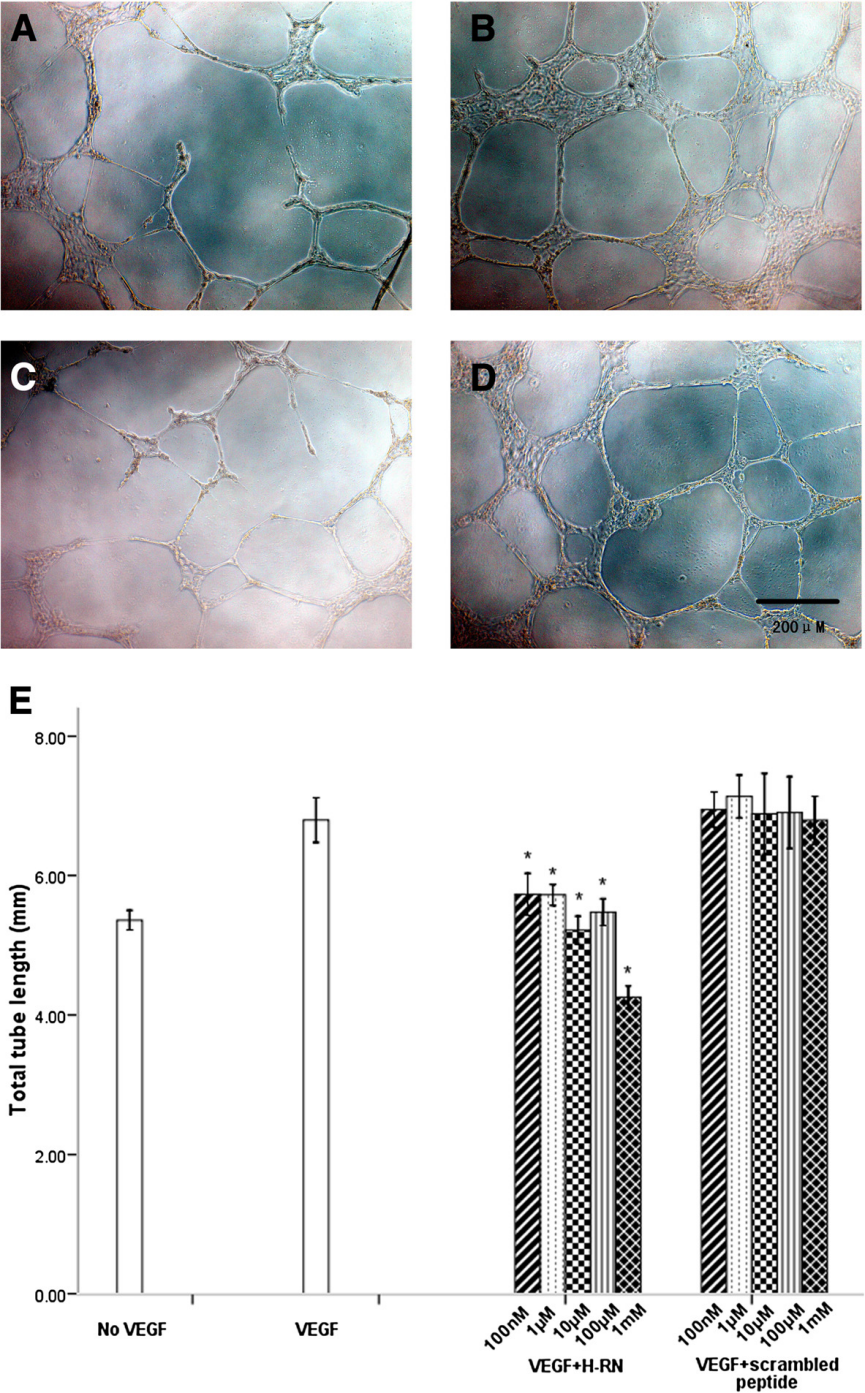
**Inhibitory effects of H-RN on HUVEC differentiation into capillary structures.** HUVECs were starved overnight and pre-incubated with different concentrations of H-RN and scrambled peptide for 30 min. VEGF (100 ng/ml) was added for peptide-treated and VEGF groups, and cells were seeded into Matrigel-coated wells. **A**, no-VEGF; **B**, 100 ng/ml VEGF; **C**, 100 ng/ml VEGF + 1 mM H-RN; **D**, 100 ng/ml VEGF + 1 mM scrambled peptide. VEGF (100 ng/ml) strongly stimulated HUVEC tube formation. H-RN significantly inhibited VEGF-stimulated HUVEC tube formation, particularly at 1mM. Scrambled peptide did not show inhibitory effects on HUVEC tube formation (magnification ×200). **E**: Quantitative analysis of tube formation under different experimental conditions using Image Program Plus software. Values represent the mean tube lengths from three independent experiments (*p<0.05, each condition versus VEGF control group).

We next assessed the ability of H-RN to affect tube formation in Matrigel. As shown in Figure 3, tube formation was strongly stimulated by VEGF (100 ng/ml). HUVEC tube formation was effectively inhibited following treatment with H-RN, especially at concentrations of 100 μM and above (*p*<0.001, Figure 3). In contrast, scrambled peptide did not inhibit VEGF-induced endothelium tube formation at any concentration (*p*>0.05, Figure 3).

### H-RN inhibits corneal angiogenesis in a mouse model

**Figure 4:**
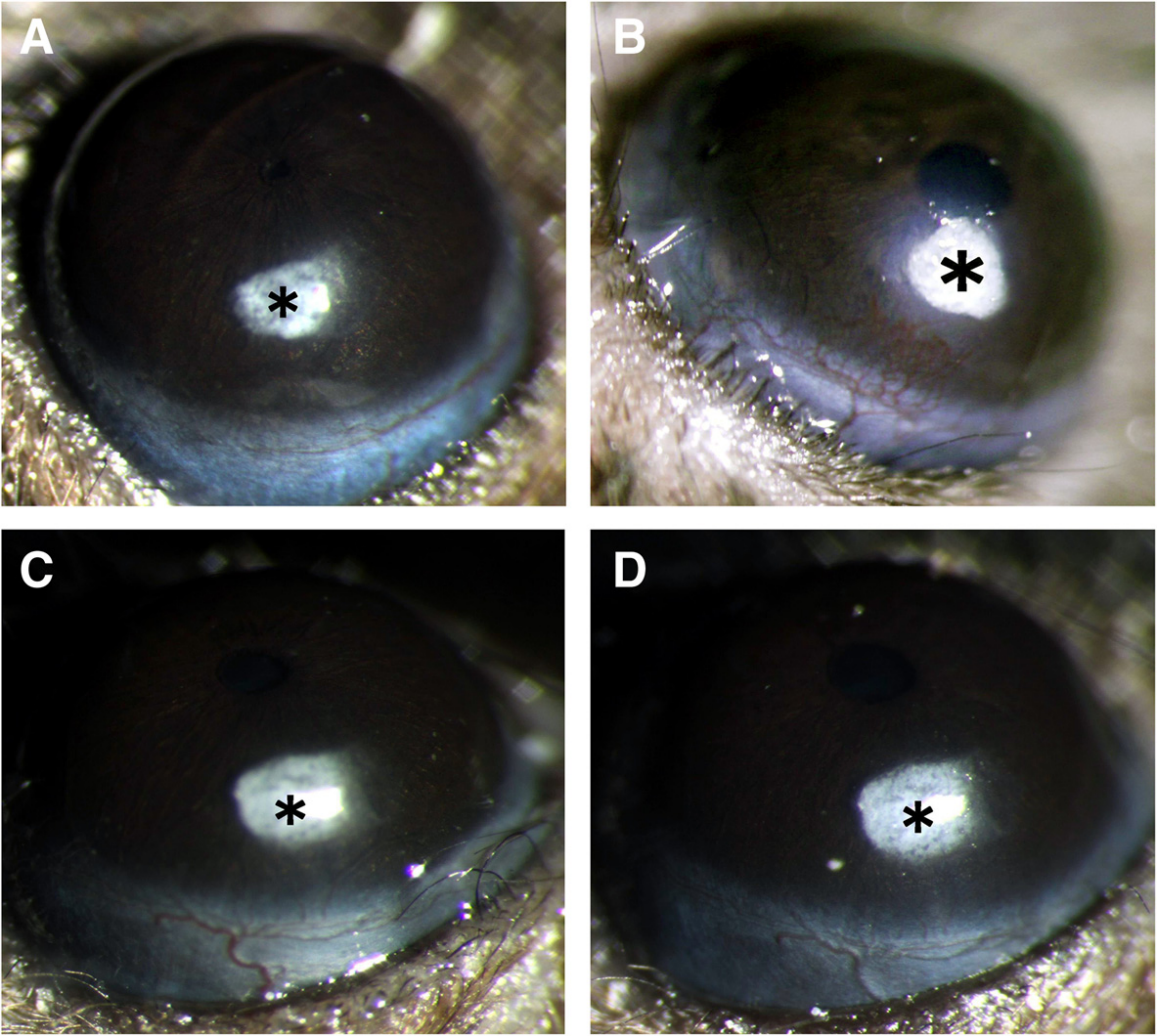
**C57BL/6 mouse corneas 7 days after implantation with pellets (asterisks) containing different reagents. A**, phosphate buffered saline (PBS); **B**, 160 ng VEGF; **C**, 160 ng VEGF and 1 μg H-RN; **D**, 160 ng VEGF and 5 μg H-RN. VEGF pellets induced a strong neovascular response, originating from the corneal limbal vessels and reaching the pellet. Pellets containing VEGF and 1 μg H-RN also induced a neovascular response, but this was less intense. Pellets containing VEGF and 5 μg H-RN resulted in a mild vascular response compared to VEGF group.

To investigate the effect of H-RN *in vivo*, we investigated the effect of H-RN on corneal angiogenesis. The growth of new capillary vessels from the corneal limbus towards implanted pellets containing VEGF or VEGF combined with peptides was assessed 7 days post-implantation. No new vessels were observed in pellets containing PBS only. Corneal neovascularization towards a pellet containing VEGF was vigorous. In contrast, neovascularization towards pellets containing both VEGF and H-RN was significantly inhibited compared to VEGF only, particularly at 5 μg H-RN (Figure 4). For quantitative analysis, the vessel area was calculated according to the maximal vessel length and clock hours. The vessel area decreased significantly in the H-RN group when compared with VEGF alone (Table 1, *p*<0.05). These results demonstrate that H-RN exhibits an inhibitory effect on VEGF-induced angiogenesis in the mouse cornea.

**Table 1:** Quantitative analysis of cornea neovascularization in different groups

**Group (n=8)**	**Maximal vessel length (mm)**	**Clock hours**	**Vessel area (mm**^**2**^**)**
PBS	0	0	0
VEGF	1.16 ± 0.27	2.24 ± 0.25	1.62 ± 0.36
1μg H-RN	0.60 ± 0.16	2.04 ± 0.51	0.79 ± 0.36*
5μg H-RN	0.40 ± 0.18	1.28 ± 0.46	0.31 ± 0.18*

* *p*< 0.05 compared to VEGF group.

### The effect of H-RN on the anti-apoptosis activity of VEGF

**Figure 5:**
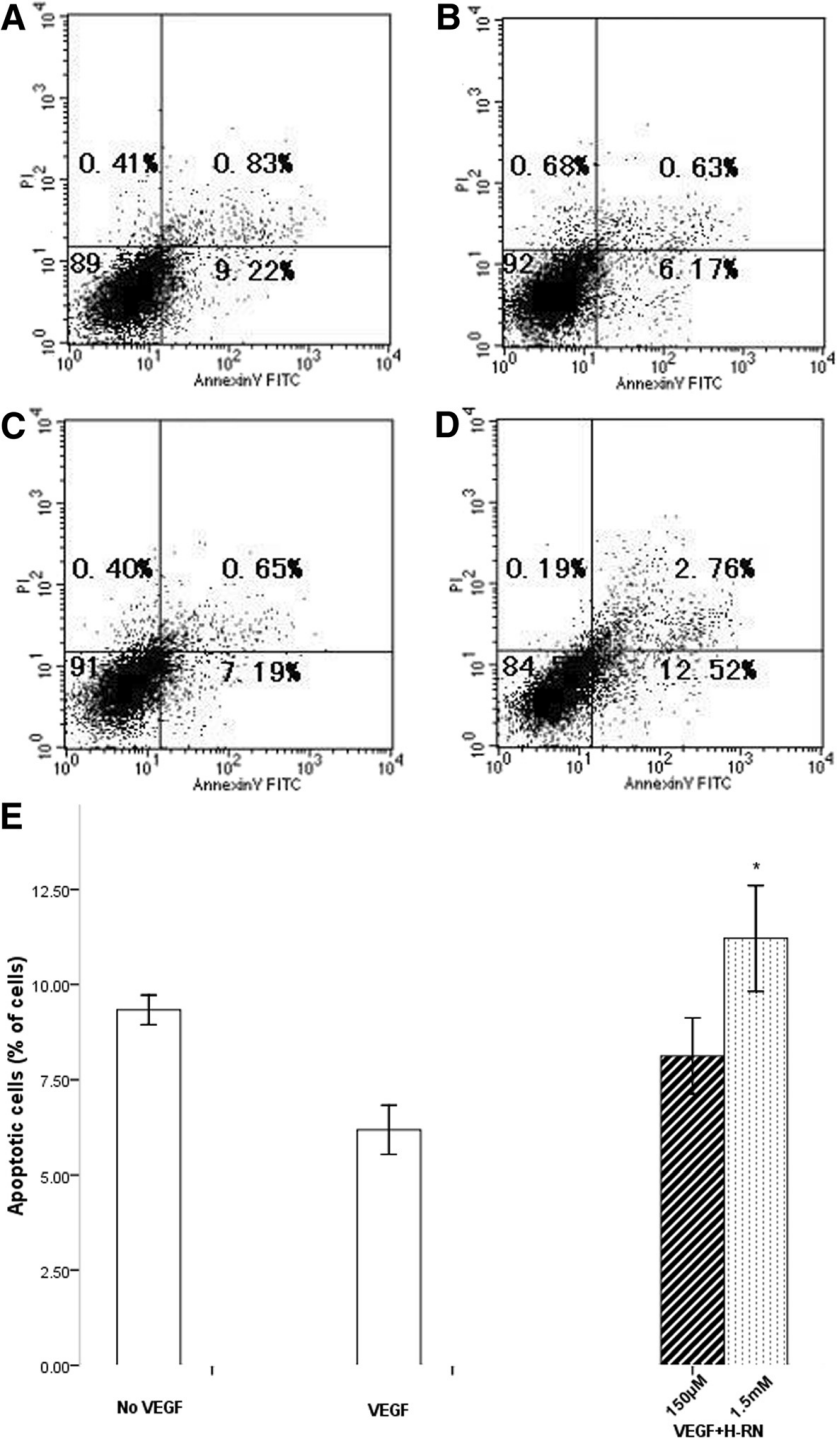
**Flow cytometry analysis of plasma membranes with annexin V-FITC / PI double staining. A**-**D**: representative photos of different groups. **A**: control. Cells were incubated in serum-free medium for 24 h following overnight starvation. The numbers in upper left, upper right, lower left and lower right quadrants represent the percentage of necrotic cells (annexin V positive, PI positive), advanced apoptotic cells (annexin V negative, PI positive), viable cells (annexin V negative, PI negative) and early apoptotic cells (annexin V positive, PI negative) respectively; **B**: VEGF. Cells were incubated in serum-free medium containing 100 ng/ml VEGF for 24 h following starvation; **C**: VEGF+150 μM H-RN. Cells were pre-incubated with 150 μM H-RN for 30 min and VEGF (100 ng/ml) was added and incubated for 24 h; **D**: VEGF+1.5 mM H-RN. Cells were pre-incubated in 1.5 mM H-RN for 30 min and VEGF (100 ng/ml) was added for 24 h; **E**: Quantitative analysis of apoptotic cells under different experimental conditions. The values represent the mean percentages of apoptotic cells in different groups. (*p<0.05, each condition versus VEGF control).

HUVECs were starved overnight and treated with various agents and apoptosis was analyzed by flow cytometry. Annexin V-FITC-/PI- cells located in the bottom left quadrant represent viable cells. Early apoptotic cells (annexin V-FITC +/PI-) located in the bottom right quadrant represent early apoptotic cells. Following treatment of HUVECs with VEGF (100 ng/ml), early apoptotic HUVECs decreased from 9.35 ± 0.27% to 4.76 ± 0.41%. However, exposure of cells to increasing concentrations of H-RN, led to an increase in the percentage of early apoptotic cells to 5.62 ± 0.14% and 9.50 ± 0.51% (*p*<0.001, versus VEGF only group) (Figure 5). These results demonstrate that VEGF protects HUVECs from apoptosis, but that H-RN may inhibit this effect.

### H-RN induces cell cycle arrest of HUVECs in G0/G1 phase

**Figure 6:**
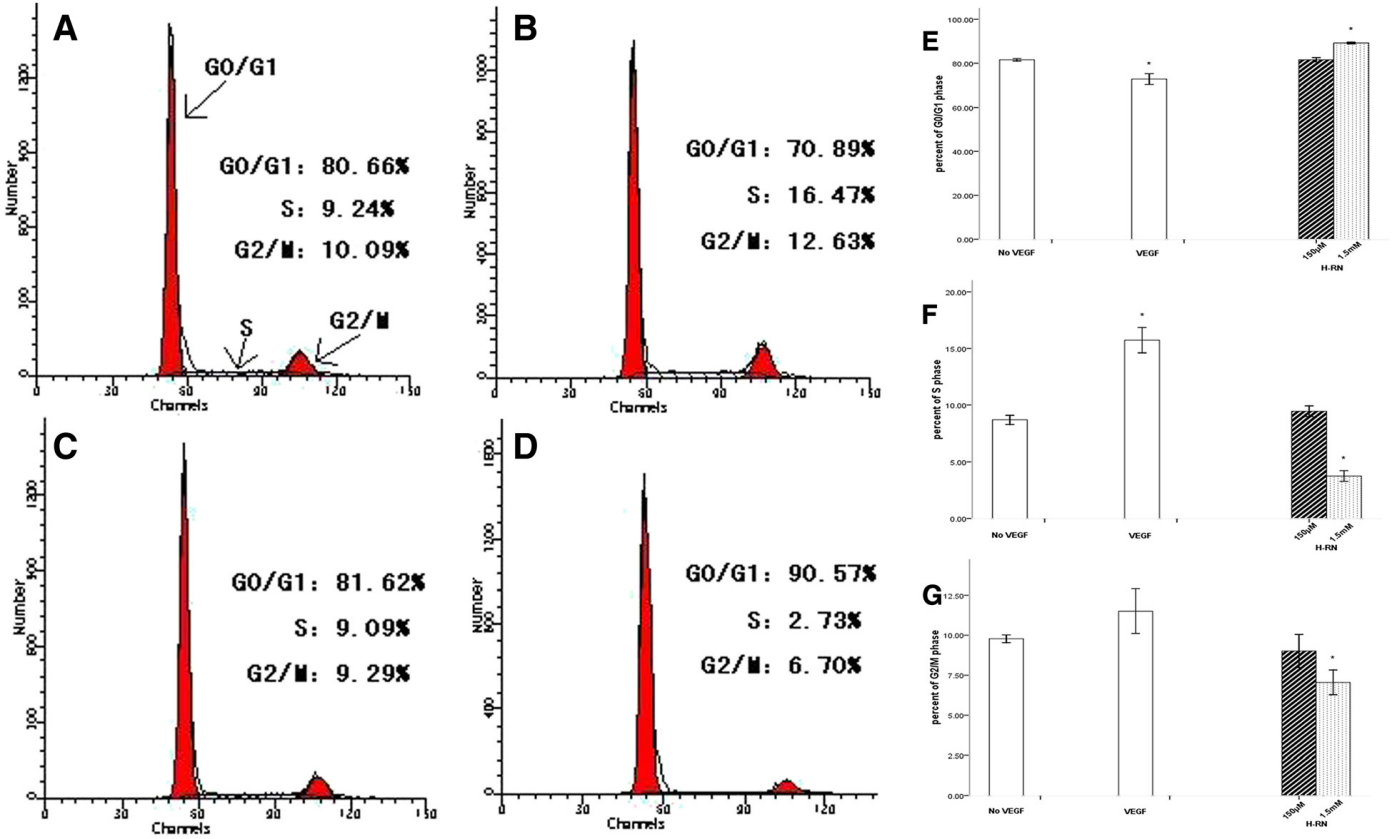
**Effect of H-RN on HUVEC cell cycle.** Cells were incubated in serum-free medium with 100 ng/ml VEGF, 150 μM H-RN or 1.5 mM H-RN for 24 h. **A-D**: Cell cycle distribution analysis by flow cytometry. Ordinate represents cell number, abscissa represents DNA content. The first sharp peak represents G0/G1 phase cells, the following lower peak represents G2/M phase cells, and the area between them represents S phase cells. The numbers on the right part of each figure represent the percentage of cells in each phase. **A**: control group; **B**: 100 ng/ml VEGF group; **C**: 150 μM H-RN group; **D**: 1.5 mM H-RN group. **E-G**: Statistical analysis among different groups. **E**: Statistical analysis of the percentage of G0/G1 phase cells among different groups; **F**: Statistical analysis of the percentage of S phase cells among different groups; **G**: Statistical analysis of the percentage of G2/M phase cells among different groups. (*p<0.05 versus no VEGF control group).

To examine the effect of H-RN on cell growth, HUVECs were incubated with VEGF or H-RN for 24 h and cell cycle was assessed by PI staining and flow cytometry. Treatment of HUVECs with VEGF significantly increased the proportion of cells in the S phase (15.73% *vs* 8.70%, *p*<0.05), and in G2/M phase (11.50% *vs* 9.78%, *p*<0.05) compared to untreated control cells. Treatment of cells with H-RN (1.5 mM) led to accumulation of cells in the G0/G1 phase (89.20% *vs* 81.53%, *p*<0.05) and a significant decrease in the proportion of cells in S phase (3.75% *vs* 8.70%, *p*<0.05) and G2/M phase, compared to control untreated cells (Figure 6).

## Discussion

Corneal neovascularization may be caused by several pathogenic conditions, including inflammatory, ischemic, degenerative or traumatic diseases of the cornea and loss of the limbal stem cell barrier. To date, numerous models of corneal angiogenesis have been developed. Thermal injury and alkali-burn models have been utilized in many studies, since these are easy to perform and corneal neovascularization may be conveniently observed [17]. Inflammation is the major mechanism of cornea angiogenesis in these models and these models were predominantly used in studies focused on inflammation. Infectious keratitis and corneal ulcers, which brought unstable experimental results and difficulties to precise quantitative analysis, are also commonly seen in these models. Mouse tumor models induce corneal neovascularization by intrastromal implantation of tumor cells in the rat or mouse cornea, where vessel growth is stimulated by tumor-secreted angiogenic factors, inflammation or immunologic mediators [18]. We previously reported the anti-angiogenic activity of H-RN in a choroid-retinal endothelial cell line (RF/6A), in the chick chorioallantoic membrane and in a mouse model of oxygen-induced retinopathy [16].

In the present study, we investigated the anti-angiogenic effect of H-RN in VEGF-induced corneal neovascularization *in vitro* using HUVECs, and *in vivo* using a mouse cornea micropocket assay, which is dependent on direct stimulation of neovascularization rather than indirect stimulation by inflammation or tumors. Our results obtained from both *in vitro* and *in vivo* models were highly reproducible and easily quantifiable [19]. In addition, VEGF was used as a direct angiogenesis stimulator in the models, thus providing meaningful results for the evaluation of an anti-VEGF and anti-angiogenic reagent.

Previously, we reported a new peptide derived from HGF, H-RN, which exhibited anti-angiogenic activity in a choroid-retinal endothelial cell line (RF/6A) and in the chick chorioallantoic membrane, as well as in a mouse model of oxygen-induced retinopathy [16]. In the present study, we investigated the anti-angiogenic activity of H-RN on corneal neovascularization. HUVECs were used for *in vitro* studies, and the effects of H-RN on VEGF-stimulated proliferation, cell migration and endothelial cell tube formation were investigated. Similar results were found as those obtained from our *in vitro* study of RF/6A cells. H-RN significantly inhibited HUVEC proliferation, migration and tube formation stimulated by VEGF. The inhibitory effects were particularly intense at high concentrations, though not dose-dependent. The scrambled peptide did not show any inhibitory effect at any concentration. In the mouse cornea micropocket assay, we found that VEGF significantly stimulated corneal angiogenesis. Neovascularization derived from the corneal limbus developed towards VEGF-containing pellets, with bushy and thick vessels migrating towards and over the surface of the white pellet. This growth was significantly inhibited following administration of H-RN. We infer that H-RN has the potential for treating pathological corneal neovascularization, and sustained release delivery may be an effective drug delivery option, although further investigation of H-RN pharmacokinetics is still required.

Li et al. [20] recently reported that topical administration of KH906, a recombinant human soluble VEGF receptor fusion protein, was capable of significantly inhibiting angiogenesis in an alkali burn corneal neovascularization rabbit model by topical administration. KH906 was administrated in three different concentrations; 5 mg/ml, 10 mg/ml and 20 mg/ml. Rabbits received topical administration (50 μl) of the different solutions four times daily for 14 days. Corneal neovascularization was analyzed 10 and 14 days after chemical cauterization. In this study, corneal neovascularization was significantly reduced in KH906-treated groups compared to control treated animals. Compared to the effective peptide quantity in our study, the total drug quantity applied in Li’s study is much higher (250 μg *vs* 5 μg), indicating that H-RN has a much lower effective concentration than KH-906. Furthermore, the treatment cycle of H-RN is significantly shorter than KH-906 (7 d *vs* 14 d), and the production cost of H-RN is also lower than KH-906. In this study, the level of VEGF in the cornea in KH906-treated groups was significantly decreased. In our study, we found that the ability of H-RN to inhibit the anti-apoptotic activity of VEGF and to induce G0/G1 phase cell cycle arrest is related to its anti-angiogenesis properties. Taken together, this demonstrates that both H-RN and KH-906 inhibit neovascularization through an anti-VEGF mechanism.

Previous reports have shown that VEGF may inhibit vascular endothelial cell apoptosis [21]. We infer that H-RN may inhibit the anti-apoptosis activity of VEGF, as demonstrated by flow cytometric analysis of apoptosis. It is well established that VEGF inhibits endothelial cells apoptosis via activation of the PI3K signaling pathway [21] and by upregulating the expression of Bcl-2 and A1 [22], important anti-apoptosis genes. Additional studies are required to determine whether H-RN inhibits activation of the PI3K signaling pathway or the expression of Bcl-2 and A1. We further investigated the effect of H-RN on the cell cycle of HUVECs, which may relate to its inhibitory effect on endothelial cell proliferation. We observed a G0/G1 phase arrest in H-RN treated cells, indicating that H-RN inhibits DNA replication of HUVECs. Cyclin, cyclin dependent kinase (CDK) [23] and cyclin dependent kinase inhibitor (CDKI) [24] are critical factors regulating the cell cycle. Future studies will aim to investigate the relationship between H-RN and these factors.

## Conclusions

In summary, we show that H-RN, a small peptide derived from HGF, displays anti-angiogenic activity in HUVECs and effectively inhibits VEGF-induced corneal angiogenesis in a mouse model. While our study indicates that the anti-angiogenic activity of H-RN is related to apoptosis and cell cycle arrest, further studies investigating the mechanisms underlying the anti-angiogenic activity of H-RN are necessary.

## Methods

### Cell culture and materials

HUVECs were obtained from the American Type Culture Collection and maintained as monolayer cultures in endothelial cell medium (ECM) supplemented with 10% fetal bovine serum (FBS) at 37°C in 5% CO_2_. Human VEGF was obtained from Sigma-Aldrich (USA). H-RN (RNPRGEEGGPW, molecular weight: 1254.34 Da) and a scrambled peptide were synthesized by solid phase peptide synthesis using an automatic peptide synthesizer (Symphony; Protein Technologies, Tucson, AZ). The end product was characterized by high-performance liquid chromatography (HPLC; LC-20A, SHIMADZU, Kyoto, Japan) and mass spectrometry (MS; Finnigan TSQ 7000; Thermo, Waltham, MA).

### Cell proliferation assay

Cell proliferation assays were performed as previously described [16,25] using the CellTiter96 AQueous One Solution Cell Proliferation Assay (MTS) kit (Promega, Madison WI, USA) according to the manufacturer’s instructions. Briefly, cells were seeded into 96-well plates (4.8×10^3^cells/well). After 24 h, cells were serum-starved overnight and treated with 100 ng/ml human VEGF (Sigma-Aldrich, USA) and increasing concentrations of H-RN (0, 1 μM, 10 μM, 100 μM or 1 mM) in 100 μL of serum free medium for 24 h. Following treatment, 20 μl of MTS reagent was added to each well and incubated for 4 h. The absorbance at 490 nm was recorded using a microplate reader (BIO-RAD, Model 680, USA). Each group was tested in triplicate and assays were repeated a minimum of three times.

### Cell migration assay

Cell migration assays were performed as previously described with modifications [16,26]. Briefly, HUVECs were starved overnight, trypsinized and suspended at a final concentration of 5×10^5^ cells/ml. Cells were pre-incubated with various concentrations of peptides for 30 min at 37°C before seeding into Transwell chambers (tissue culture treated, 10 mm diameter, 8.0 μm pores; Corning Inc. New York, N.Y., USA), Cells (5×10^4^) were seeded onto the upper Transwell chamber and VEGF (100 ng/ml) was placed into the lower chamber. The assembled cell culture insert chamber was then incubated at 37°C for 24 h. After removal of non-migrating cells in the upper chambers with a cotton swab, migrated cells on the lower surface of the porous membrane were fixed, stained with Gram’s stain and photographed under a light microscope (Olympus, Tokyo, Japan). Five random fields (×200) were chosen in each insert and the cell number was quantified manually. Each experiment was repeated 3 times.

### Endothelial cell tube formation assay

Tube formation assays to assess the formation of vascular-like structures by HUVECs on growth factor-reduced Matrigel (BD Biosciences) were performed as previously described [16,27]. Cells (2.5×10^4^) were pre-incubated with various concentrations of peptides (100 nM – 1 mM), seeded into 96-well culture plates pre-coated with Matrigel in serum free medium containing VEGF (100 ng/ml) and incubated at 37°C for 18 h. Tube formation was observed using an inverted phase contrast microscope (Olympus, Tokyo, Japan). Images were captured with a digital camera (Olympus, Tokyo, Japan). The degree of tube formation was quantified by measuring the length of tubes in three randomly chosen low power fields (×100) from each well using the Image-Pro Plus Program (version 5.1, Media Cybernetics, Inc. America). Each group was tested in triplicate. Each experiment was repeated 3 times.

### Corneal neovascularization model

Male C57BL/6J mice (7-9 weeks old) were obtained from the Shanghai Laboratory Animal Centre, Chinese Academy of Sciences. All experiments were consistent with the ARVO Statement for the Use of Animals in Ophthalmic and Vision Research. The animals were divided into four groups. A corneal micropocket assays were performed as previously described on one eye of each mouse [19,28] using pellets comprising the slow-release polymer Hydro (polyhydroxyethylrnethacrylate [polyHEMA]) and sucralfate. 0.5 μl PBS containing 160 ng VEGF or not was added on to each pellet. Pellets containing VEGF were then resuspended with 1 μg or 5 μg of peptides, and these pellets were only used in VEGF treated group, while pellets containing PBS were used in control group. All pellets were dried under sterile air before storage in 4°C. Mice were anesthetized with 2% chloralhydrate and an intrastromal pocket was surgically created in the cornea. A slow release pellet was inserted into the intrastromal pocket (VEGF, control, 1 μg peptides, 5 μg peptides; n=8 mice per group, 32 mice total). Ofloxacin ointment was applied to the operated eye in order to release the irritation and prevent infection. On post-operative day 7, mice were anesthetized, and the corneas were microscopically examined using an Olympus SZX2-ILLT stereoscope. Corneal neovascularization was evaluated by measuring the maximal vessel length from limbal vasculature toward the pellet (R1; in mm) and the contiguous circumferential zone of neovascular clock hours (R2). The neovascular area was calculated by the formula: Area (mm^2^) = 0.5×3.14×R1×R2×0.4 (mm).

### Flow cytometry assay

HUVECs were seeded in 6-well plates (6×10^4^ cells/well). After overnight starvation, cells were incubated with VEGF (100 ng/ml) in ECM culture medium (Gibco, USA). For treatment with peptides, cells were pre-incubated with H-RN (150 μM or 1.5 mM) or scrambled peptide for 30 min prior to addition of VEGF (100 ng/ml). Cells were incubated for 24 h and apoptosis was analyzed by annexin V-FITC / propidium iodide (PI) staining according to the manufacturer’s instructions (Invitrogen, USA). Briefly, cells were rinsed with ice-cold PBS and resuspended at a final concentration of 2-5×10^5^/ml in 250 μl binding buffer. Five microliters of annexin V-FITC stock solution was added to the cells and rinsed for 3 minutes at 4°C. Then 10μl PI (20μg/ml) was added and incubated in the dark at room temperature for 10 min. Cells were analyzed by flow cytometry (FACS Calibur, BD Biosciences, Franklin Lanes, NJ, USA) equipped with Cell Quest software. For each sample, approximately 1×10^4^ cells were analyzed [29].

### Cell cycle analysis

HUVECs were seeded on 6-well plates and incubated with or without 100ng/ml VEGF 24 h after starvation overnight. In peptides treated group, cells were incubated with 150 μM or 1.5mM H-RN for 24 h. HUVECs were harvested, washed with PBS and fixed with 70% ethanol for 30 min at 4°C. Cells were washed once with PBS, and incubated in PBS containing 50 μg/ml PI, 200 μg/ml RNase A and 0.1% Triton for 30 min at 37°C in dark. Cells were analyzed by flow cytometry (FACS Calibur, BD Biosciences, Franklin Lanes, NJ, USA), and the data analysis was performed using Cell Quest software [30,31].

### Statistical analysis

For experiments with four treatment groups and various treatment concentrations, univariate analysis of variance was used. For comparison of the differences between groups, a posthoc LSD test was used. All values are expressed as the mean ± SD. An alpha level of <0.05 was used as the criterion of significance.

## Abbreviations

HGF K1: Hepatocyte growth factor kringle 1 domain; VEGF: Vascular endothelial growth factor; HUVECs: Human umbilical vein endothelial cells; ECM: Endothelial cell medium; FBS: Fetal bovine serum; HPLC: High-performance liquid chromatography; polyHEMA: Polyhydroxyethylrnethacrylate; PBS: Phosphate Buffer Solution

## Competing interests

The authors declare that they have no competing interests.

## Authors’ contribution

YS carried out the majority of experiments, contributed to the final preparation of the manuscript and to the analysis of data. LS and ZW contributed to the experiments. YX designed and contributed to the preparation of the manuscript. XX contributed to the design of the experiments,supervised the experiments and contributed to the preparation of the manuscript. All authors read and approved the final manuscript.
